# Impact of fear of coronavirus disease 2019 on attention-deficit/hyperactivity disorder traits associated with depressive symptoms, functional impairment, and low self-esteem in university students: a cross-sectional study with mediation analysis

**DOI:** 10.1265/ehpm.24-00230

**Published:** 2025-01-10

**Authors:** Tomoko Suzuki, Toshiyuki Ohtani, Michiko Nakazato, Ariuntuul Garidkhuu, Basilua Andre Muzembo, Shunya Ikeda

**Affiliations:** 1 Department of Public Health, School of Medicine, International University of Health and Welfare, Chiba, Japan; 2 Safety and Health Organization, Chiba University, Chiba, Japan; 3 Department of Psychiatry, School of Medicine, International University of Health and Welfare, Chiba, Japan; 4 Research Institute of Nursing Care for People and Community, University of Hyogo, Akashi, Japan

**Keywords:** ADHD, COVID-19, Depressive symptoms, Functional impairment, Mediation analysis, Self-esteem, Student

## Abstract

**Background:**

High levels of attention-deficit/hyperactivity disorder (ADHD) traits are associated with various outcomes, including depressive symptoms, functional impairment, and low self-esteem. Additionally, individuals with high levels of ADHD traits are reported to be more adversely affected by fear of coronavirus disease 2019 (COVID-19). The current study aimed to examine whether the association between ADHD traits and outcomes was partially mediated by fear of COVID-19 using mediation analysis.

**Methods:**

A cross-sectional study was conducted in a sample of university students in medical-related faculties (n = 1,166). ADHD traits, fear of COVID-19, depressive symptoms, functional impairment, and self-esteem were assessed using the adult ADHD Self-Report Scale, Fear of COVID-19 Scale, K6 Scale, Sheehan Disability Scale, and Rosenberg Self-Esteem Scale, respectively. We used linear regression analysis and the Paramed command in Stata to analyze whether fear of COVID-19 mediated the association between ADHD traits and outcomes.

**Results:**

ADHD traits were significantly associated with outcomes. Regarding the impact of fear of COVID-19, the results revealed a significant association between ADHD traits and fear of COVID-19, and between fear of COVID-19 and outcomes. The results of the mediation analyses showed that the association between ADHD traits and outcomes was partially mediated by the fear of COVID-19 (depressive symptoms: direct effect B = 1.029, 95% confidence interval [CI] 0.878, 1.181, indirect effect B = 0.021, 95% CI 0.002, 0.040; functional impairment: direct effect B = 0.786, 95% CI 0.593, 0.979, indirect effect B = 0.033, 95% CI 0.005, 0.060; self-esteem: direct effect B = −1.052, 95% CI −1.226, −0.878, indirect effect B = −0.024, 95% CI −0.046, −0.002).

**Conclusions:**

Developing preventive measures against the adverse impacts of pandemics like COVID-19 will be particularly important for individuals with high levels of ADHD traits in future.

**Supplementary information:**

The online version contains supplementary material available at https://doi.org/10.1265/ehpm.24-00230.

## Background

Attention-deficit/hyperactivity disorder (ADHD) is a chronic neuropsychiatric disorder with core symptoms of inattention, hyperactivity, and impulsivity, which can cause severe impairment and morbidity [1]. ADHD traits are associated with functional impairment in several life domains [1–3] and can lead to difficulty concentrating, difficulty completing tasks, confusion, impatience, and impulsive behavior. The consequences of these traits may ultimately affect an individual’s ability to function at work, school, home, and in social environments. For example, regarding academic ability, ADHD traits are associated with academic impairment [4]. Similar results have been reported in studies of workers and university students who are not diagnosed with ADHD but have high levels of ADHD traits [5, 6]. Numerous negative experiences of functional impairment can negatively affect self-esteem, and individuals with ADHD have been reported to exhibit lower levels of self-esteem compared with non-ADHD controls [7, 8]. Furthermore, ADHD in adults is often concurrent with other psychiatric diseases, including depression [9, 10]. A survey of college students who were not diagnosed with ADHD revealed a significant association between ADHD symptoms and depression [11]. Psychiatric diseases may exacerbate impairments in daily life and can have a negative impact on self-esteem.

The coronavirus disease 2019 (COVID-19) pandemic has had widespread international impacts since the end of December 2019. During this period, the COVID-19 pandemic has been a significant stress factor, negatively affecting mental health and causing fear, panic, and mental distress [12]. Fear is one of the most common psychological reactions to disease pandemics [13] and potentially affects mental health among the general population. A meta-analysis indicated that the COVID-19 pandemic increased the prevalence of mental health problems, including depression, anxiety, distress, and insomnia [14]. Furthermore, fear of COVID-19 emerged as a major psychological problem during the COVID-19 pandemic [15]. Additionally, fear of COVID-19 was positively associated with COVID-19-related functional impairment (work/school, social, and home life) [16]. Individuals with pre-existing conditions, such as ADHD, are considered to be at particular risk of adverse effects related to the COVID-19 pandemic [17]. One previous study reported that the pandemic has exacerbated ADHD symptoms and concurrent difficulties [18]. In a study of the negative impact of fear of COVID-19, individuals with ADHD were found to be more likely to exhibit fear and negative feelings regarding the risk of infection [19].

**Fig. 1:**
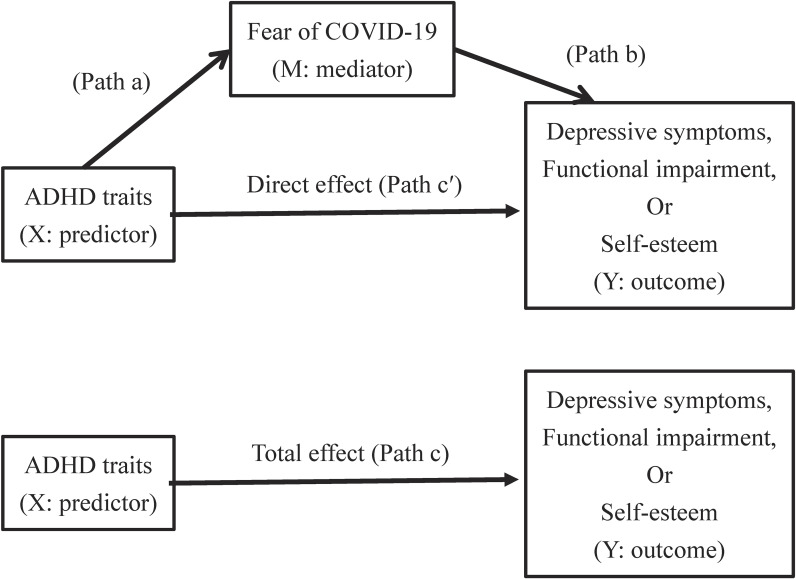
Mediation model as a directed acyclic graph Model with fear of COVID-19 as a mediator of the effect of ADHD traits on depressive symptoms, functional impairment, and self-esteem.

The findings discussed above suggest that high levels of ADHD traits are associated with depressive symptoms, functional impairment, and low self-esteem. Individuals with higher levels of ADHD traits have been reported to be more adversely affected by fear of COVID-19 [19]. Because of the adverse impacts of the fear of COVID-19, individuals with high levels of ADHD traits may experience more depressive symptoms, more functional impairment, and lower self-esteem. It is possible that there is a direct effect on the relationship between ADHD traits and outcomes (depressive symptoms, functional impairment, and low self-esteem), and an indirect effect mediated by COVID-19 on the relationship between ADHD traits and outcomes. However, the indirect effects of fear of COVID-19 are currently unclear. Because it has been widely reported that ADHD is highly comorbid with other psychiatric disorders, such as depression [9–11], the primary aim of this study was to clarify the mediating effect of fear of COVID-19 on the association between ADHD traits and depressive symptoms. The secondary aim was to clarify the mediating effect of fear of COVID-19 on the association between ADHD traits and functional impairment, and between ADHD traits and low self-esteem. Mediation analyses were conducted using ADHD traits as continuous variables, as ADHD traits are continuously distributed in the general population [20]. We hypothesized that higher levels of ADHD traits would be more likely to be associated with these negative outcomes, in part, via fear of COVID-19. Figure 1 shows the conceptual framework of the present study, as a directed acyclic graph (DAG) of our mediation analysis. In this DAG, X (ADHD traits) represents the predictor and the exposure variable, Y (depressive symptoms, functional impairment, and low self-esteem) represents the outcome variable, and M (Fear of COVID-19) represents the mediator. Arrows indicate assumed causal effects. The path X → M → Y represents the indirect effect (Path a and Path b), and the path X → Y represents the direct effect (Path c′). The total effect is the sum of all pathways (direct and indirect, Path c).

The participants in this study were students in medical-related faculties, who were exposed to a stressful environment involving intensive lectures and examinations. Medical students have been reported to experience high rates of depression, and the prevalence of depressive symptoms among medical students is reported to be higher than that in the general population [21, 22]. Therefore, medical students represent a group with a high need for preventing the negative impacts of stressors.

## Methods

### Participants

This cross-sectional study was conducted as a survey of university students in medical-related faculties in a major university. We collected data three times, in 2019, 2020, and 2021. The 2021 data set was used for the present analyses because the Fear of COVID-19 Scale was administered in 2021. In 2021, 1,231 students participated in the survey between May 2021 and March 2022. Of these, 65 students were excluded from analyses because of missing data in the data types used for the present analysis, which left 1,166 study participants. Participants were surveyed using a self-administered questionnaire. Information was collected on various characteristics, including age, sex, school year, and faculty.

### Measures

The adult ADHD Self-Report Scale (ASRS) [23] was used to determine participants’ ADHD traits. The Japanese version of the ASRS was used in this study [24]. The ASRS is an 18-item self-report scale covering the 18 symptoms of adult ADHD from the Diagnostic and Statistical Manual of Mental Disorders, Fourth Edition (DSM-IV) [25]. The ASRS Screener is a short version of the ASRS that includes six of the 18 ASRS questions. The six-item shortened form has been previously validated as a screening measure for the presence of adult ADHD [23]. Each ASRS question asks how often a particular ADHD symptom has occurred in the past 6 months. Total possible scores range from 0 to 6, with 0 corresponding to no ADHD symptoms and 6 corresponding to extremely severe symptoms. An ASRS score of 4 or higher, which is considered to reflect suspected ADHD [23], was defined as high ADHD traits.

The Fear of COVID-19 Scale (FCV-19S) [13] was designed to assess individuals’ fear towards COVID-19. We used the Japanese version of the FCV-19S [26]. The FCV-19S contains seven items that specifically ask about fear caused by COVID-19, and each item is measured on a 5-point Likert scale. Total possible scores range from 7 to 35. The higher the score, the greater the fear of COVID-19.

Depressive symptoms were measured using the K6 scale [27]. We used the Japanese version of the K6 scale [28]. The K6 scale consists of six items assessing the frequency with which respondents had experienced symptoms of psychological distress (e.g., “feeling so sad that nothing can cheer you up”) during the preceding 30 days. The response options range from 0 (none of the time) to 4 (all of the time); total possible scores range from 0 to 24. Higher scores indicate more severe mental disorders. Depressive symptoms were defined in the present study when subjects had a K6 score of 9 or higher [28].

Functional impairment in adults with mental illness was measured using the Sheehan Disability Scale (SDS) [29]. We used the Japanese version of the SDS [30]. The SDS is useful for monitoring functional impairment in adults with ADHD [31]. The SDS is a three-item test measuring the impact of symptomatology on work/school work, social and family functioning. Each item is rated from 0 to 10. The sum of all item scores yields the global SDS score, which ranges from 0 to 30. Higher scores on the SDS indicate greater impairment. An SDS score of 1 or higher was defined as functional impairment in the present study.

Self-esteem scores were calculated by using the Rosenberg Self-Esteem Scale (RSES) [32]. We used the Japanese version of the RSES [33]. RSES evaluates global self-esteem, on the basis of positive and negative beliefs and perceptions about oneself. It comprises 10 items for evaluating individual self-esteem with a four-point Likert scale. RSES scores are presented in a range of 10–40 points. The higher the score, the higher the level of self-esteem. The lowest tertile RESE score was defined as low self-esteem in the present study.

### Statistical analyses

Continuous variables are presented as the mean ± standard deviation, and categorical variables are presented as percentages. All statistical analyses were performed using Stata 17 with all available updates as of April 2023 (StataCorp, College Station, Texas 77845, USA). We calculated Cronbach’s alpha and Pearson’s correlation coefficients for ADHD traits, fear of COVID-19, depressive symptoms, functional impairment, and self-esteem scores. Using linear regression analysis, mediation analysis [34] was performed to assess whether the association between ADHD traits (continuous) and outcomes (depressive symptoms, functional impairment, and self-esteem) was partially mediated by the fear of COVID-19 (Fig. 1). According to Baron and Kenny’s criteria [34], four statistical conditions need to be met to establish complete mediation or partial mediation. First, ADHD traits (independent variable) should significantly influence depressive symptoms, functional impairment, and self-esteem (dependent variable, path c: total effect). Second, ADHD traits (independent variable) should significantly influence the fear of COVID-19 (mediator, path a). Third, the fear of COVID-19 should also significantly influence depressive symptoms, functional impairment, and self-esteem when adjusted for ADHD traits (path b). Fourth, mediation occurs if when Path a and Path b are controlled, the significant association between ADHD traits and depressive symptoms, functional impairment, and self-esteem is attenuated or becomes non-significant (path c′: direct effect). Complete mediation is established if the association between ADHD traits and depressive symptoms, functional impairment, and self-esteem is reduced to zero (path c′). The analysis was conducted to test the significance of the indirect effects (path ab). The product “a × b” quantifies the indirect effect. The analysis was carried out using the Paramed command in Stata [35], following the Baron and Kenny method [34]. Paramed simultaneously estimates direct, indirect, and total effects from a single mediation model [35, 36]. The total effect is the sum of direct and indirect effects, making these conceptually linked [35, 36]. The multivariate-adjusted model was adjusted for age, sex, school year, and faculty (medicine, nursing, and other). Regression coefficients with 95% confidence intervals (CIs) were reported. Similarly, estimates for the direct, indirect, and total effects were provided. For categorical variable analysis, logistic regression analysis was used to calculate odds ratios (OR) for outcomes (depressive symptoms, functional impairment, and low self-esteem) in individuals with high ADHD traits (ASRS score ≥ 4) compared with those in individuals with low ADHD traits (ASRS score < 3). The multivariate-adjusted model was adjusted for age, sex, school year, and faculty (medicine, nursing, and other). Two-sided p-values less than 0.05 were considered to be statistically significant.

### Ethics statement

The Ethics Committee of the International University of Health and Welfare reviewed and approved this study (No. 19-Im-008), which was conducted in accordance with the standards specified in the 1964 Declaration of Helsinki. All participants provided written informed consent prior to enrollment in the study.

## Results

Participants’ characteristics are shown in Table 1. In total, 1,166 students participated in this study. Distributions of all variables of interest are shown in Supplementary Fig. 1. All measures have satisfactory internal consistency reliability, with Cronbach’s alpha coefficients of 0.75 or higher (Table 2). Pairwise correlations for all the variables of interest are shown in Table 2. Fear of COVID-19 scores, depressive symptoms scores, and functional impairment scores were significantly positively associated with ADHD trait scores, and self-esteem scores were significantly negatively associated with ADHD trait scores. Depressive symptom scores or functional impairment scores were significantly positively associated with fear of COVID-19 scores, and self-esteem scores were significantly negatively associated with fear of COVID-19 scores.

**Table 1:** Participants’ characteristics (N = 1,166)

Age	(years)	20.8 ± 2.9
Sex	Male	445 (38.2%)
School year (grade)	1	305 (26.2%)
	2	287 (24.6%)
	3	281 (24.1%)
	4	221 (19.0%)
	5	72 (6.2%)
Faculty	Medicine	507 (43.5%)
	Nursing	374 (32.1%)
	Other	285 (24.4%)

Mean ± SD, n (%)

**Table 2:** Internal consistency (Cronbach’s α), and Pearson’s correlations for all variables of interest (N = 1,166)

	**Variables**	**Mean ± SD**	**Cronbach’s alpha**	**1**	**2**	**3**	**4**	**5**
1	ADHD traits, ASRS score	2.3 ± 1.5	0.75	1				
2	Fear of COVID-19, FCV-19S score	13.1 ± 4.9	0.84	0.09**	1			
3	Depressive symptoms, K6 score	3.4 ± 4.1	0.88	0.37***	0.13***	1		
4	Functional impairment, SDS score	4.2 ± 5.1	0.85	0.22***	0.13***	0.57***	1	
5	Self-esteem, RSES score	25.9 ± 4.8	0.86	−0.35***	−0.13***	−0.46***	−0.27***	1

**p < 0.01, ***p < 0.001

ADHD, attention-deficit/hyperactivity disorder; ASRS, adult ADHD Self-Report Scale; FCV-19S, Fear of COVID-19 Scale; SDS, Sheehan Disability Scale; RSES, Rosenberg Self-Esteem Scale.

The mediating effects of fear of COVID-19 through ADHD traits on depressive symptoms, functional impairment, and self-esteem in students are shown in Table 3, using Baron and Kenny’s method. The results of the multivariate-adjusted model are shown below. Path a: there was a significant association between ADHD traits and fear of COVID-19 (B = 0.275, 95% CI: 0.084 to 0.466). Path b: fear of COVID-19 was significantly positively associated with depressive symptoms and functional impairment (B = 0.076, 95% CI: 0.031 to 0.122, and B = 0.119, 95% CI: 0.061 to 0.177, respectively), and was significantly negatively associated with self-esteem (B = −0.088, 95% CI: −0.141 to −0.036). Path c′: direct effect of ADHD traits on depressive symptoms and functional impairment was significantly positive (B = 1.029, 95% CI: 0.878 to 1.181, and B = 0.786, 95% CI: 0.593 to 0.979, respectively), and direct effect of ADHD traits on self-esteem was significantly negative (B = −1.052, 95% CI: −1.226 to −0.878). Path c: total effect of ADHD traits on depressive symptoms and functional impairment was significantly positive (B = 1.050, 95% CI: 0.898 to 1.203, and B = 0.819, 95% CI: 0.626 to 1.012, respectively), and the total effect of ADHD traits on self-esteem was significantly negative (B = −1.076, 95% CI: −1.251 to −0.902). Path ab: indirect effect of the association between ADHD traits and depressive symptoms and functional impairment via the fear of COVID-19 was significantly positive (B = 0.021, 95% CI: 0.002 to 0.040, and B = 0.033, 95% CI: 0.005 to 0.060, respectively), and the indirect effect of the association between ADHD traits and self-esteem via the fear of COVID-19 was significantly negative (B = −0.024, 95% CI: −0.046 to −0.002). Each indirect effect was small but significant. Regarding the significant association between ADHD traits and depressive symptoms, functional impairment, and self-esteem, the direct effect was attenuated compared with the total effect. The results suggest that the association between ADHD traits and the outcome (depressive symptoms, functional impairment, and self-esteem) was partially mediated by fear of COVID-19.

**Table 3:** Mediating effect of COVID-19 fear through ADHD traits on outcomes (N = 1,166)

**Predictor variable**	**Mediator variable**	**Outcome variable**	**Path**	**Effect**	**Crude model**			**Adjusted model**		

					**B**	**B 95% CI**	**p**	**B**	**B 95% CI**	**p**
(Common to all three models)										
ADHD traits	Fear of COVID-19		a		0.311	(0.122–0.501)	0.001	0.275	(0.084–0.466)	0.005
(Model with depressive symptoms as outcome)										
	Fear of COVID-19	Depressive symptoms	b		0.078	(0.033–0.123)	0.001	0.076	(0.031–0.122)	0.001
ADHD traits		Depressive symptoms	c′	Direct	1.013	(0.862–1.163)	<0.001	1.029	(0.878–1.181)	<0.001
ADHD traits		Depressive symptoms	c	Total	1.037	(0.887–1.188)	<0.001	1.050	(0.898–1.203)	<0.001
ADHD traits	Fear of COVID-19	Depressive symptoms	a × b	Indirect	0.024	(0.004–0.045)	0.020	0.021	(0.002–0.040)	0.032
(Model with functional impairment as outcome)										
	Fear of COVID-19	Functional impairment	b		0.118	(0.060–0.176)	<0.001	0.119	(0.061–0.177)	<0.001
ADHD traits		Functional impairment	c′	Direct	0.709	(0.516–0.902)	<0.001	0.786	(0.593–0.979)	<0.001
ADHD traits		Functional impairment	c	Total	0.746	(0.552–0.939)	<0.001	0.819	(0.626–1.012)	<0.001
ADHD traits	Fear of COVID-19	Functional impairment	a × b	Indirect	0.037	(0.008–0.066)	0.012	0.033	(0.005–0.060)	0.021
(Model with self-esteem as outcome)										
	Fear of COVID-19	Self-esteem	b		−0.097	(−0.149–−0.044)	<0.001	−0.088	(−0.141–−0.036)	0.001
ADHD traits		Self-esteem	c′	Direct	−1.102	(−1.276–−0.927)	<0.001	−1.052	(−1.226–−0.878)	<0.001
ADHD traits		Self-esteem	c	Total	−1.132	(−1.307–−0.957)	<0.001	−1.076	(−1.251–−0.902)	<0.001
ADHD traits	Fear of COVID-19	Self-esteem	a × b	Indirect	−0.030	(−0.055–−0.005)	0.017	−0.024	(−0.046–−0.002)	0.032

Mediation analysis was performed using linear regression analysis. B: unstandardized regression coefficient; 95% CI: 95% confidence interval; Adjusted model: Adjusted for age, sex, school year, and faculty; ADHD, Attention-deficit/hyperactivity disorder (continuous). ADHD traits, fear of COVID-19, depressive symptoms, functional impairment, and self-esteem were assessed using ASRS, FCV-19S, K6 Scale, SDS, and RSES, respectively.

Table 4 shows the ORs for outcomes (depressive symptoms, functional impairment, and low self-esteem) in individuals with high ADHD traits compared with those in individuals with low ADHD traits. The results of the analysis after adjustment for possible confounding factors (Model A) were as follows. Students with high ADHD trait scores were significantly more likely to report depressive symptoms (OR = 3.96, 95% CI: 2.76 to 5.67), functional impairment (OR = 2.57, 95% CI: 1.84 to 3.59), and low self-esteem (OR = 2.71, 95% CI: 2.02 to 3.66), compared with students with low ADHD traits. In Model B, with depressive symptoms as the outcome, after adjusting for the variables in Model A plus an additional adjustment variable (functional impairment), students with high ADHD trait scores and those with functional impairment were significantly more likely to report depressive symptoms (OR = 3.19, 95% CI: 2.20 to 4.63, and OR = 9.80, 95% CI: 5.05 to 19.00, respectively). In Model B, with functional impairment as the outcome, after adjusting for the variables in Model A plus an additional adjustment variable (depressive symptoms), students with high ADHD trait scores and those with depressive symptoms were significantly more likely to report functional impairment (OR = 2.03, 95% CI: 1.43 to 2.88, and OR = 9.90, 95% CI: 5.10 to 19.20, respectively). These results suggest that the association between ADHD traits and depressive symptoms is likely to be distinct from the association between ADHD traits and functional impairment. However, it is important to note that these associations may potentially influence each other.

**Table 4:** Outcomes associated with ADHD traits (N = 1,166)

**Outcome**	**Crude model**		**Adjusted model A**		**Adjusted model B**		

	**OR (95% CI)**	**p**	**OR (95% CI)**	**p**	**Additional adjustment variable**	**OR (95% CI)**	**p**
Depressive symptoms	4.02 (2.81–5.75)	<0.001	3.96 (2.76–5.67)	<0.001		3.19 (2.20–4.63)	<0.001
					Functional impairment	9.80 (5.05–19.00)	<0.001
Functional impairment	2.44 (1.76–3.39)	<0.001	2.57 (1.84–3.59)	<0.001		2.03 (1.43–2.88)	<0.001
					Depressive symptoms	9.90 (5.10–19.20)	<0.001
Low self-esteem	2.79 (2.08–3.73)	<0.001	2.71 (2.02–3.66)	<0.001			

Logistic regression analysis was used to calculate odds ratios (OR) for outcomes (depressive symptoms, functional impairment, and low self-esteem) in individuals with high ADHD traits compared with those in individuals with low ADHD traits. Definitions: High ADHD traits: ASRS score ≥ 4; Low ADHD traits: ASRS score < 3; Depressive symptoms: K6 score ≥ 9; Functional impairment: SDS score ≥ 1; Low self-esteem: RESE score ≤ 24 (lowest tertile). Adjusted model A: Adjusted for age, sex, school year, and faculty; Adjusted model B: adjusted for variables in model A plus additional adjustment variable; OR: odds ratio, 95% CI, 95% confidence interval; ADHD, Attention-deficit/hyperactivity disorder; ASRS, adult ADHD Self-Report Scale; FCV-19S, Fear of COVID-19 Scale; SDS, Sheehan Disability Scale; RSES, Rosenberg Self-Esteem Scale.

## Discussion

This cross-sectional study was conducted as a survey of university students (n = 1,166) in medical-related faculties, including medical students who are at high risk for depression [21, 22]. ADHD traits were significantly associated with depressive symptoms, functional impairment, and low self-esteem. Regarding the impact of fear of COVID-19, there was a significant association between ADHD traits and fear of COVID-19, and there was a significant association between fear of COVID-19 and depressive symptoms, functional impairment, and low self-esteem. As hypothesized, the results of mediation analyses showed that the associations between ADHD traits and depressive symptoms, functional impairment, and low self-esteem were partially mediated by fear of COVID-19. Although COVID-19 has been reported to exacerbate difficulties exhibited by individuals with high levels of ADHD traits, no previous studies have reported that this association is partially mediated by fear of COVID-19. Our results, which were partially mediated by fear of COVID-19, revealed that individuals with higher levels of ADHD traits were more affected by fear of COVID-19 than individuals with lower levels of ADHD traits. This indicates that the effects of fear of COVID-19 caused individuals with higher ADHD traits to be more likely to experience worse outcomes (depressive symptoms, functional impairment, and low self-esteem) compared with individuals with lower ADHD traits, except for negative outcomes that are directly attributable to ADHD traits.

Regarding the fear of COVID-19 for individuals with high levels of ADHD traits, the present results revealed a significant association between ADHD traits and fear of COVID-19. Although few previous studies have examined the impact of fear of COVID-19, individuals with ADHD were reported to be more likely to show fear of COVID-19, and differences in pandemic-related anxiety may be caused by emotion regulation difficulties in individuals with ADHD [19]. Additionally, several studies reported that individuals with high levels of ADHD traits were more adversely affected by the COVID-19 pandemic, but fear of COVID-19 was not examined [18, 37]. Individuals with ADHD are particularly vulnerable to the negative impacts of COVID-19. The COVID-19 pandemic has been reported to exacerbate ADHD symptoms and co-occurring difficulties [18]. Untreated ADHD is a risk factor for acquiring COVID-19, possibly because the inattentive, hyperactive, and impulsive traits of individuals with ADHD tend to place individuals in high-risk situations that can be particularly stressful [37]. Additionally, COVID-19 infection in patients with ADHD was found to be associated with more severe symptoms [38]. Therefore, individuals with high levels of ADHD traits may be more afraid of the risk of contracting COVID-19.

Our finding of a significant association between ADHD traits and depressive symptoms is consistent with previous reports [9, 10]. Importantly, the results of our mediation analyses examining the impact of the COVID-19 pandemic indicated that the association between ADHD traits and depressive symptoms was partially mediated by the fear of COVID-19. To the best of our knowledge, no previous studies of this topic have used mediation analyses. Fear of COVID-19 was found to cause stronger emotional responses in young people with ADHD compared with their peers in a previous study [19], potentially suggesting that COVID-19 acted as a mediator. In a previous study investigating impacts of the COVID-19 pandemic other than the effects of COVID-19-related fear, depression and school dropout rates were reported to increase during the COVID-19 pandemic for adolescents or young adults with ADHD [39]. Additionally, COVID-19-related restrictions were found to result in more severe low mood and isolation in young people with ADHD compared with pre-pandemic conditions [40].

Our finding of a significant association between ADHD traits and functional impairment is consistent with previous reports [1, 3]. By examining the impact of the COVID-19 pandemic, our mediation analysis results revealed that the association between ADHD traits and functional impairment was partially mediated by the fear of COVID-19. To the best of our knowledge, no previous studies have used mediation analyses to examine the effects of fear of COVID-19. However, several studies reported that the effects of the COVID-19 pandemic exacerbate functional impairment in individuals with high levels of ADHD traits, consistent with the current results [18, 19, 41]. Disruption of life activity by the COVID-19 pandemic, particularly an increase in remote learning, may exacerbate ongoing impairments associated with ADHD [19]. The disruption to daily life associated with the COVID-19 pandemic has been reported to significantly exacerbate the functional impairment already experienced by individuals with ADHD [18, 41]. Additionally, children and young people with ADHD were reported to have experienced a deterioration in functional impairment during the COVID-19 pandemic [42].

Our finding of a significant association between ADHD traits and low self-esteem is consistent with previous reports [7, 8]. In examining the impact of the COVID-19 pandemic, the results of the mediation analyses showed that the association between ADHD traits and low self-esteem was partially mediated by the fear of COVID-19. We were unable to find previous studies reporting this effect. However, this result is theoretically plausible because the COVID-19 pandemic has exacerbated ADHD symptoms and accompanying difficulties [18]. Additionally, ADHD traits are reported to be associated with poor academic achievement [4]. Negative experiences can affect the formation of an individual’s self-esteem, and poor academic achievement is likely to result in low self-esteem. Individuals with ADHD have been reported to exhibit lower levels of self-esteem compared with non-ADHD controls [7, 8].

Participants in this study were university students in medical-related faculties exposed to stressful environments, including intensive lectures and examinations. Depressive symptoms are reported to be more prevalent among medical students than in the general population [21, 22]. Additionally, during the COVID-19 pandemic, a meta-analysis reported that the prevalence of mental problems among medical students was high, including depression (41%), stress (34%), and burnout (38%) [43]. Conversely, prior to the COVID-19 pandemic, the prevalence of depression and depressive symptoms among medical students was reported to be 24% [22], indicating that the prevalence of depression during the COVID-19 pandemic was notably higher than pre-pandemic levels. The major risk factors during the COVID-19 pandemic include exposure to COVID-19, fear of infection, academic stress, economic trouble, fear of education impairment, online learning trouble, loneliness, low social support, and young age [43]. Among them, a particular risk factor for students in medical-related faculties may be their heightened fear of COVID-19 infection. This anxiety stems from their curriculum, which includes clinical training involving direct patient contact. In addition, academic stress may also be a major risk factor for students because of intensive lectures and examinations. Therefore, the target participants in this study constitute a group requiring particularly attentive care.

### Implications

The current findings have several important implications that should be considered. The mediation analysis results revealed that the associations between ADHD traits and depressive symptoms, functional impairment, and self-esteem were partially mediated by the fear of COVID-19. Several previous studies have reported that individuals with high levels of ADHD traits were more adversely affected by the COVID-19 pandemic [18, 37]. However, no previous studies conducted mediation analyses to determine which factors related to COVID-19 led to worse outcomes for individuals with high levels of ADHD traits.

### Limitations

The current study involved several potential limitations that should be considered. First, because our study used a cross-sectional design, we were unable to reveal causal relationships between variables. Future research should aim to clarify the causal relationships between variables using longitudinal study designs. Second, the self-administered questionnaires applied in this study to assess ADHD traits, fear of COVID-19, depressive symptoms, functional impairment, and self-esteem only identified trends and symptoms, and clinical conditions were not diagnosed. Third, in the present study, only fear of COVID-19 was considered as an impact of COVID-19. Several previous studies have examined impacts of COVID-19 other than fear of COVID-19, and future studies should consider whether other COVID-19-related factors also act as mediating factors. Fourth, while other paths are theoretically possible for our hypothesis, in the current study, we opted for the simplest and most likely path.

## Conclusions

The current cross-sectional study was conducted in a sample of university students (n = 1,166) in medical-related faculties. ADHD traits were significantly associated with depressive symptoms, functional impairment, and low self-esteem. Regarding the impact of fear of COVID-19, the results revealed a significant association between ADHD traits and fear of COVID-19, and a significant association between fear of COVID-19 and depressive symptoms, functional impairment, and low self-esteem. The results of the mediation analyses showed that the associations between ADHD traits and depressive symptoms, functional impairment, and low self-esteem were partially mediated by the fear of COVID-19. In future, individuals with high levels of ADHD traits have a particular need for prevention against the adverse impacts of new pandemics like COVID-19.

## Abbreviations

95% CI: 95% confidence interval
ADHD: Attention-deficit/hyperactivity disorder
ASRS: adult ADHD Self-Report Scale
COVID-19: coronavirus disease 2019
DSM-IV: Diagnostic and Statistical Manual of Mental Disorders
FCV-19S: Fear of COVID-19 Scale
SDS: Sheehan Disability Scale
RSES: Rosenberg Self-Esteem Scale

## Supplementary information

**Additional file 1:** **Supplementary Fig. 1.** Distributions of all variables of interest. ADHD, Attention-deficit/hyperactivity disorder; ASRS, Adult ADHD Self-Report Scale; FCV-19S, Fear of COVID-19 Scale; SDS, Sheehan Disability Scale; RSES, Rosenberg Self-Esteem Scale.

## Declarations

### Availability of data and materials

Data available on request from authors.
