# Supplementary material for: Impact of fear of coronavirus disease 2019 on attention-deficit/hyperactivity disorder traits associated with depressive symptoms, functional impairment, and low self-esteem in university students: a cross-sectional study with mediation analysis
Source: Environ Health Prev Med. 2025 Jan 10;30:2. doi: 10.1265/ehpm.24-00230 (PMC11744025; doi:10.1265/ehpm.24-00230)
Supplement: Supplementary file 1 — Additional file 1: Supplementary Fig. 1. Distributions of all variables of interest. ADHD, Attention-deficit/hyperactivity disorder; ASRS, Adult ADHD Self-Report Scale; FCV-19S, Fear of COVID-19 Scale; SDS, Sheehan Disability Scale; RSES, Rosenberg Self-Esteem Scale. [file ehpm-30-002-s001.docx]

**Supplementary Fig. 1**


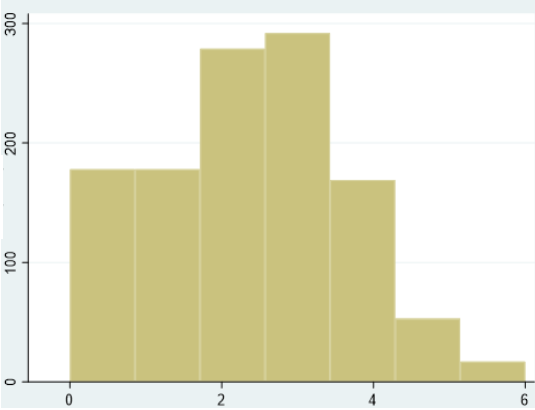


No. of subjects

ADHD traits, ASRS score


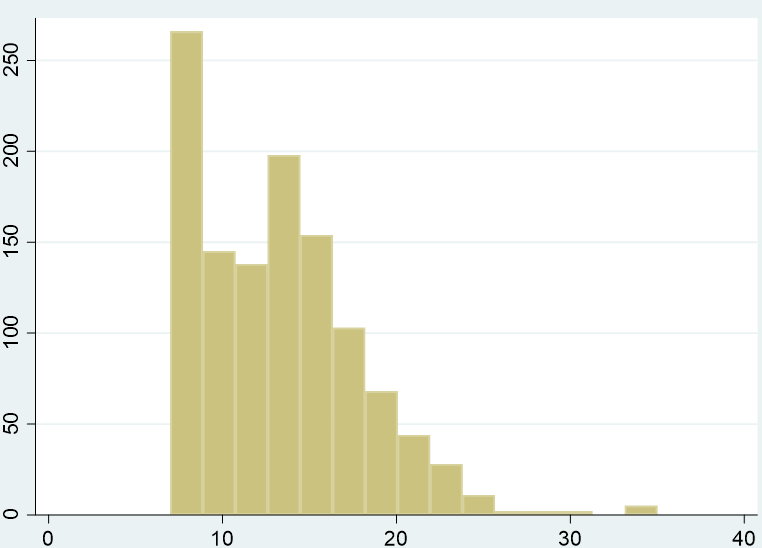


No. of subjects

Fear of COVID-19, FCV-19S score

Depressive symptoms, K6 score


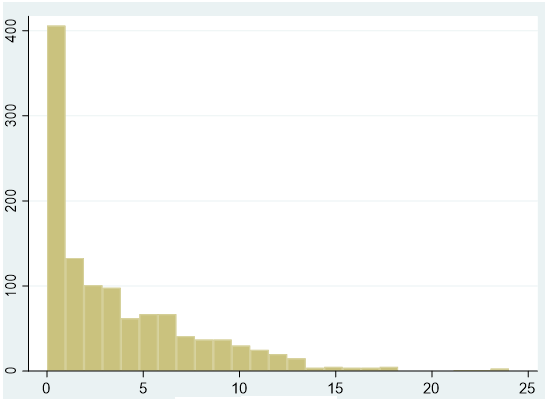


No. of subjects


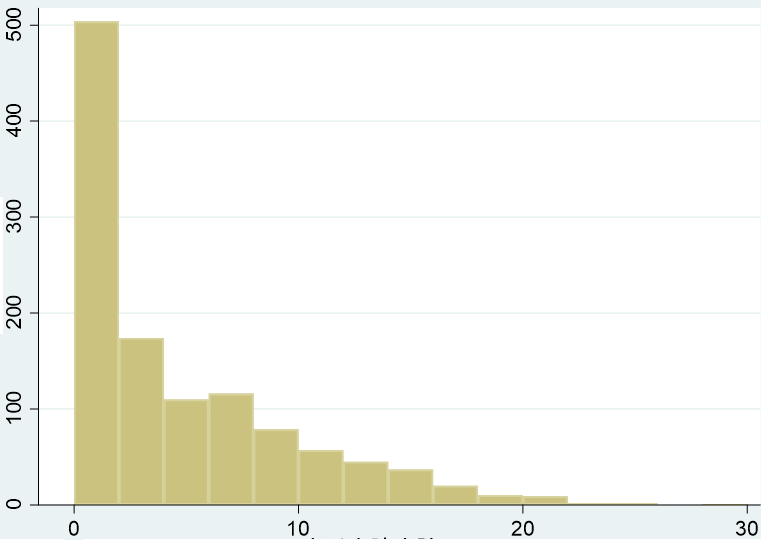


No. of subjects

Functional impairment, SDS score


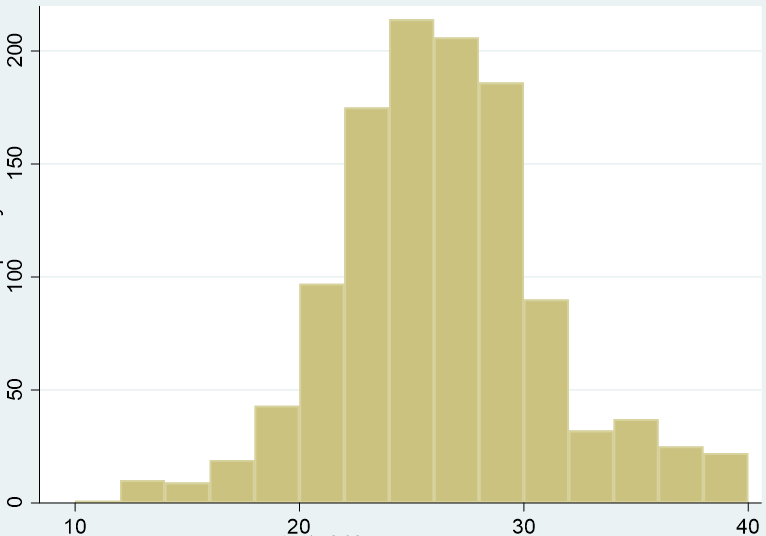


No. of subjects

Self-esteem, RSES score
